# Autologous Fat Grafting Improves Facial Nerve Function

**DOI:** 10.1155/2015/520746

**Published:** 2015-06-08

**Authors:** Marco Klinger, Andrea Lisa, Fabio Caviggioli, Luca Maione, Matteo Murolo, Valeriano Vinci, Francesco Maria Klinger

**Affiliations:** ^1^Reconstructive and Aesthetic Plastic Surgery School, Department of Medical Biotechnology and Translational Medicine (BIOMETRA), University of Milan, Plastic Surgery Unit, Humanitas Research Hospital, Rozzano, 20089 Milan, Italy; ^2^Reconstructive and Aesthetic Plastic Surgery School, University of Milan, MultiMedica Holding S.p.A., Plastic Surgery Unit, Sesto San Giovanni, 20099 Milan, Italy

## Abstract

We describe the case of a 45-year-old male patient who presented a retractile and painful scar in the nasolabial fold due to trauma which determined partial motor impairment of the mouth movements. We subsequently treated him with autologous fat grafting according to Coleman's technique. Clinical assessments were performed at 5 and 14 days and 1, 3, and 6 months after surgical procedure and we observed a progressive release of scar retraction together with an important improvement of pain symptoms. A second procedure was performed 6 months after the previous one. We observed total restoration of mimic movements within one-year follow-up. The case described confirms autologous fat grafting regenerative effect on scar tissue enlightening a possible therapeutic effect on peripheral nerve activity, hypothesizing that its entrapment into scar tissue can determine a partial loss of function.

## 1. Introduction

Trauma to the facial nerve is the second most common cause of facial paralysis and can result in devastating consequences including ocular complications, impaired speech, feeding difficulties, and inability to convey emotions through facial expression [1].

Its management depends on several factors: if the facial paralysis appears immediately or later after trauma, the extent of paralysis (complete or incomplete), the type of trauma (blunt, penetrating, or iatrogenic), the condition of the nerve, the duration of facial paralysis, and the status of the motor end plates of the facial muscles.

Medical therapy and close observation are appropriate for those patients with incomplete paralysis, delayed onset of paralysis, blunt trauma to the extratemporal nerve, and injury occurring on the medial to the lateral canthus of the eye [2].

Autologous fat grafting is a relatively new technique which has been recently adopted to treat various pathologic conditions in reconstructive surgery. In particular, our group applied its regenerative properties to treat hypertrophic scars and burn keloid obtaining scar release and quality improvement [3, 4].

Moreover, excellent results were described for the treatment of pain syndromes such as postmastectomy pain syndrome (PMPS) [5]. We also successfully applied autologous fat grafting in the treatment of Arnold neuralgia [6], a chronic headache of cervical origin (both chronic cervicogenic and occipital neuralgia), caused by cicatricial entrapment of the great occipital nerve.

In the present report, we describe the case of a 45-year-old male patient who presented with a scar in the nasolabial fold due to a trauma and consequent partial motor impairment of the mouth movements treated with autologous fat grafting.

## 2. Case Presentation

A healthy 45-year-old male patient came to our Department of Plastic Surgery. He referred a scar placed in the right cheek, proximal to the nasolabial fold, which determines a partial impairment to mimic expression, together with pain and cosmetic discomfort, during both movements and rest, and cosmetic discomfort. Trauma occurred 3 years before clinical evaluation with a hypertrophic scar in the depth palpable since almost 2 years.

His medical, family, personal, and previous clinical histories were unremarkable.

On close inspection, we observed a 5 cm long linear hypertrophic scar in the right cheek proximal to the nasolabial fold. Mouth movements were conserved except for a partial limit to mimic movements and his ability to smile.

The patient referred pain during digital pressure.

Moreover, we noticed a less prominence of the nasolabial fold probably due to a reduction of the underlying support of the tissue medial to the crease and descent in a similar fashion to the lateral tissue.

After collection of both clinical history and examination, we proposed our patient surgical scar tissue correction with autologous fat grafting.

Our patient was informed about surgical procedure, in particular regarding autologous fat grafting unpredictable reabsorption rate and clinical results which aimed to improve scar cosmetic appearance.

**Figure 1:**
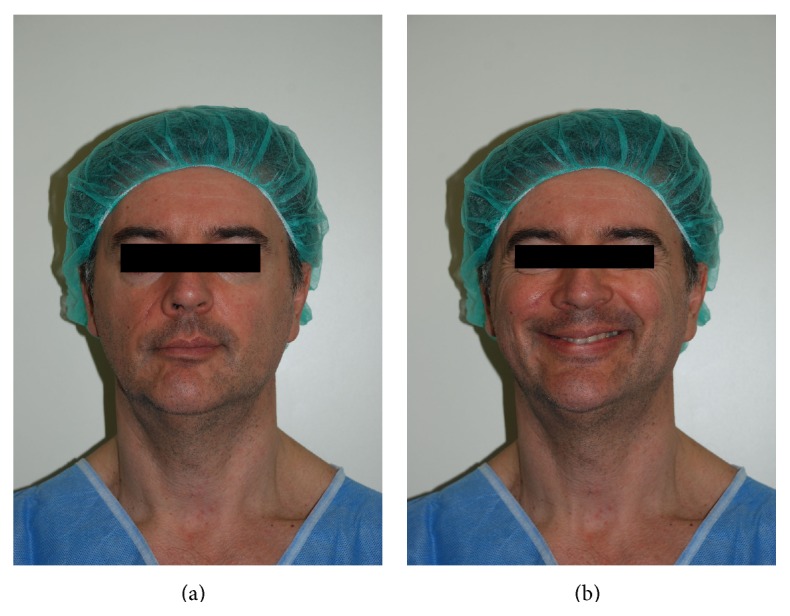
(a, b) Frontal view of our patient who presented with painful posttraumatic scar in the nasolabial fold. In (b), the partial motor impairment of the mouth movements can be enlightened.

Both informed consent form and preoperative images were collected (Figures 1(a) and 1(b)).

After routine preoperative examination and clinical assessment, the patient underwent liposuction under sedation and local anesthesia. The adipose tissue was harvested from the right flank, which is an easy accessible and abundant reservoir of adipose tissue. Following Coleman's procedure [7], the obtained fat was processed by centrifugation at 3000 rpm for 3 minutes. The fat graft was then injected using an 18-gauge angiographic needle with a snap-on wing (Cordis, Johnson & Johnson Company, N.V., Roden, Netherlands) in the scar area. The lysis of scar tissue was obtained by moving the needle in an anterograde direction and leveraging the strength of exiting fat to overcome the fibrous tissue resistance and a retrograde technique that is performed entering the needle for its entire length at the dermal-epidermal junction and then, while extracting the needle, releasing fat. In both ways, we obtained a subcision of the scar to release entrapped nerves. A total of about 7 cc of adipose tissue was injected.

Following surgery pressure dressing was applied over donor site for 5 days and antibiotic therapy was recommended for 5 days.

Clinical assessment was subsequently performed after surgical procedure at 5 and 14 days and 1, 3, and 6 months. During all clinical follow-ups, we observed a progressive release of scar retraction together with an important improvement of pain symptoms. Therefore, we proposed a new procedure 6 months after the first one. During the second procedure, we injected 6 cc of adipose tissue.

**Figure 2:**
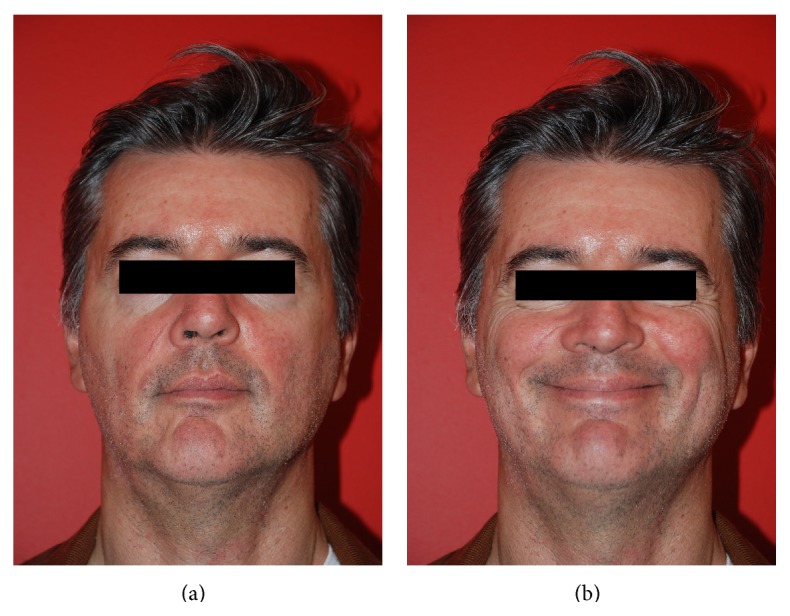
(a, b) Frontal view after one-year follow-up. After 2 sessions of autologous fat grafting pain sensation was resolved and we obtained scar release. In (b), the total restoration of mimic movements is evident.

Three months after the second procedure that we conducted with similar technique, we observed further increase of scar release. Pain sensation was resolved and in addition a total restoration of mimic movements was evident within a one-year follow-up (Figures 2(a) and 2(b)).

No local or systemic signs of infection were found; no complications occurred.

## 3. Discussion

Facial nerve injury can be a devastating injury resulting in functional deficits and psychological distress although the nerve is anatomically intact or the injury is limited to the medial portion of a peripheral branch.

In such cases, treatment is usually medical with close observation and possible electrophysiologic testing to monitor progression. Unfortunately, these treatments determine only partial results.

In our case, the patient presented with a posttraumatic scar in the right nasolabial fold with pain sensation and partial impairment to mimic movements probably due to damage to the buccal and zygomatic branches of the facial nerve which supply motor innervation to the upper lip.

We opted for a treatment with autologous fat graft in order to improve scar appearance and reduce pain, relying on experience in the treatment of multiple pathological status beyond all scar treatment [8] and pain syndromes [9].

In our experience, in the treatment of burn scars, we observed at histologic examination patterns of new collagen deposition, local hypervascularity, and dermal hyperplasia in the treated specimens.

Moreover, our clinical experience in the field of postmastectomy pain syndrome treatment showed a therapeutic effect of autologous fat grafting to reduce neuropathic pain.

Our group treated also chronic headaches of cervical origin, both chronic cervicogenic and occipital neuralgia. These cervical headache syndromes generally present with myofascial spasm and local, scar-like entrapment of the occipital nerve by local fibrosis and adherences, conditions which represent a continuous trigger for nerve excitatory pattern.

One of the etiologic theories to explain autologous fat grafting effect is based on the nerve pathways intraoperative damage and nerves entrapment in scar fibrosis, which represents a continuous trigger for nerve excitation.

For these reasons, we consider autologous fat grafting to be an innovative solution for pain syndromes related to scar retraction.

The case presented adds an interesting aspect that has never been described before.

After treatment, our patient referred scar release, cosmetic improvement, and pain reduction, together with a total recovery of facial movements.

These findings could widen our knowledge about autologous fat grafting regenerative effects.

In fact, we have already hypothesized that autologous fat graft, because of its regenerative role, could promote reorganization of fibrotic tissue together with soft tissue regeneration, leading to scar release and reducing nerve excitatory pattern with consequent positive clinical results on pain control, but we never observed any effect on nerve function as in the present case.

Our experience could be explained supposing a regenerative effect also on peripheral nerve function hypothesizing that its entrapment into scar tissue can determine a partial loss of function.

In the described case, it can be postulated that buccal and zygomatic branches of the facial nerve were entrapped into scar tissue and the release determined by fat grafting has determined a correction of nerve function loss.

Despite the unclear underlying mechanism and the lack of histological evidences, our satisfactory and unexpected result supports our experience and our choice of treating posttraumatic facial scar with partial loss of nerve function.

Although we reported a single case based only on observation without more specific proofs such as histologic proof and electrophysiologic testing, it could bring evidences which can widen our knowledge on autologous fat grafting regenerative effect on nerve function.
